# Data for stable formulation of steroid hormone receptor-targeted liposomes for cancer therapeutics

**DOI:** 10.1016/j.dib.2016.01.003

**Published:** 2016-02-27

**Authors:** Priyanka Sharma, Rajkumar Banerjee, Kumar Pranav Narayan

**Affiliations:** a Department of Biological Sciences, Birla Institute of Technology and Science Pilani, Hyderabad Campus, Telangana 500078, India; b Biomaterials Group, CSIR-Indian Institute of Chemical Technology, Hyderabad, Telangana 500007, India

**Keywords:** Steroid hormone, Liposomes, Lipoplexes, Anti-cancer gene, Transfection, Cytotoxicity

## Abstract

A detailed description of steroid hormone ligand containing liposomes and their stability has been given. Liposomes were complexed with β-gal DNA and used to transfect cancer and non-cancer cells. The stability of the liposomes and lipoplexes were analysed using dynamic light scattering and DNA-binding gel images. The formulations were used to assess the delivery of anticancer gene, p53 in cancer cells. The dataset consists of DNA-binding gel images, transfection, cytotoxicity and reverse transcriptase PCR images.

Specifications table

**Table:** 

Subject area	*Biology*
More specific subject area	*Cancer therapeutics*
Type of data	*Gel image, text file*
How data was acquired	*Zeta sizer (Malvern, Australia) ELx ELISA reader (Biotech)*
Data format	*Raw, analyzed*
Experimental factors	*Cells were pretreated with antagonist (RU486 & Eplerenone) and Mineralocorticoid receptor siRNA*
Experimental features	*Liposomes and lipoplexes formation*
*Size and charge on liposomes*
*Transfection of cells with lipoplexes*
*Cytotoxicity assessment*
*siRNA down-regulation*
Data source location	*NA*
Data accessibility	*Data is with this article*

Value of the data

• Targeting diseased tissue/organ is important as most of the therapies lead to toxic effects on normal tissues.
• The method can be used for stable cationic liposome preparation and efficient transfection of cells.
• The data presents a novel way to treat receptor linked diseases.

## 1 Data

The liposomal formulation contains mineralocorticoid receptor specific ligand, spironolactone (hence the name SP for the targeted formulation) alongside the cationic lipid DODEAC and co-lipid cholesterol. The size and potential i.e. stability of the liposomes were done using dynamic light scattering at 0 day, after 4 days, 8 days and 12 days as shown in Tables S1 & S2. The stability of the complexes were analysed by DNA-binding studies after 30 min, 4 h and 24 h of complex formation (Fig. S2). The targeted liposomes(SP) and non-targeted liposomes (DO) were used to transfect cells of both cancer (Fig. S1) and non-cancer origin. The transfection efficiency was observed after down-regulation of the receptor with antagonist. The cells were down-regulated with mineralocorticoid receptor specific MRsiRNA and the transfected with the lipoplexes to see the effect of receptor down-regulation on transfection efficiency and cytotoxicity (Figs. S3 & S4).

## 2 Experimental design, materials and methods

### 2.1 Preparation of Liposomes

Liposomes were prepared by the method described by [1]. Thin lipid films were prepared by drying the chloroform solution of 1 µM of DODEAC, 1 µM of cholesterol and three different concentrations of spironolactone i.e. 1 µM, 0.75 µM and 0.50 µM for three different liposomal formulation i.e. 1:1:1, 1:1:0.75, 1:1:0.50 under a gentle stream of nitrogen and dried in vacuum for at least 4 h. It was hydrated with 1 ml of sterile water overnight and then first vortexed to remove any adhering lipid film, then subjected to a low intensity bath sonication for 15 min at room temperature and finally probe sonication for 2 min using a constant duty cycle and output control magnitude of 2–3 Branson Sonifier 450.

### 2.2 Preparation of lipoplex and its treatment to cells

The lipid-DNA complex or lipoplex was prepared according to the protocol described by [5]. Briefly, for in vitro studies related to toxicity and gene transfection studies, liposomes were serially diluted in serum free media in final volume of 50 ml and to it fixed amounts of pDNA (0.3 µg/well of 96 well plates) diluted in 50 ml of serum free media were added. The charge ratios of cationic lipid to DNA were maintained as 1:1,2:1, 4:1 and 8:1. The mixtures were shaken in room temperature for 15 min following which 10% serum containing media (200 ml) were added to each mixture. Finally, 100 ml of resulting solution was then added to triplicate wells of cell containing 96-well plate. For RT-PCR and western blot studies, the amount of pDNA used per well in 6-well plates was 2 µg and was lipoplexed in a cationic lipid to DNA(±) charge ratio of 4:1.

### 2.3 Gel retardation of lipoplexes

The DNA binding ability of the cationic lipids were assessed by gel retardation assay on a 1% agarose gel. 0.3 μg of *p*CMV- SPORT-β-gal was complexed with cationic lipids (1:1:0.75, molar ratios were mixed with DNA at final charge ratios of 8:1, 4:1, 2:1 and 1:1) in a total volume of 25 μL in Hepes buffer, pH 7.4 and incubated at room temperature for 20–25 min. 4 μL of 6X loading buffer (0.25% bromo phenol blue, 40% sucrose) was added to it and 20 μL of the resultant solution was loaded in each well. The lipoplexes were run on 1% agarose gel, electrophoresed at 80 V for approximately 2 h and the DNA bands were visualized by ultraviolet illumination. To check the stability at different time points, lipoplexes were run on 1% agarose gel after 4 h and 24 h of complex formation (Fig. S2).

### 2.4 Determination of Zeta potential, particle size, and polydispersity index of liposomes

Values of the Zeta potential of liposomes indirectly reflect vesicle surface net charge and can therefore be used to evaluate the extent of interaction of the liposomal surface cationic charges with the anionic charges of DNA. The average particle size and the polydispersity of the particle-size distribution of the liposomes were determined by Zeta sizer. Zeta potential is an indirect measurement of the vesicle surface charge, and it can be used to evaluate the extent of interaction of the liposomal surface cationic charges with the anionic charges of DNA [2], [3], [4]. To check the stability of the liposomes, size and potential of the liposomes were checked every 4 days.

### 2.5 Gene transfections with β-gal plasmid

Cells were seeded at a density of 10,000 cells per well in a 96-well plate usually 18–24 h before transfection. Transfection of cell was performed by method described by [1]. 0.3 µg of β-gal (diluted to 50 µl with plain DMEM) was complexed with varying amount of cationic liposomes (diluted to 50 µl with plain DMEM) for 15–20 min. The molar ratios (lipid:DNA) were 8:1, 4:1, 2:1, 1:1. After formation of lipoplexes, 200 µl of DMEM containing 10% FBS (CMIX) was added to the resulting lipoplexes for triplicate experiments. Cells were washed with phosphate buffer saline (PBS), pH 7.4 (1×100 µl) and then treated with lipoplex (100 µl). After incubation of the cells at a humified atmosphere containing 5% CO_2_ at 37 °C for 4 h, the media was completely removed and 100 µl of DMEM containing 10%FBS (CMIX) were added to the cells. The reporter gene activity was assayed after 48 h. The media were completely removed from the wells and the cells were lysed with 50 µl of 1X lysis buffer (0.1% NP-40) for 25–30 min. The cell lysates were assayed by taking the β-galactosidase readings and comparing the protein content of them with that of BSA.

For RU486 and Eplerenone pretreated experiments, cells were treated with RU486 and Eplerenone (in dimethyl sulphoxide) at a final concentration of 100 µmol/lt for 2 h [5]. Cells were incubated at a humified atmosphere containing 5% CO_2_ at 37 °C. Media were removed and the cells were washed with PBS (1×100 µl). The cells were subsequently treated with the lipoplexes and reporter gene assay was performed according to the above-mentioned procedure. The transfection values were reported as the average values of the triplicate experiment performed in the same plate on the same day. To verify reproducibility, each transfection experiment was performed at least 4 times.

### 2.6 Cytotoxicity studies

Cytotoxicities of the liposomes were evaluated by the 3-(4, 5-dimethylthiazol-2-yl)−2, 5-diphenyltetrazolium bromide (MTT) reduction assay [6]. Briefly, cells were seeded at a density of 10,000 cells/well in a 96-well plate usually 18–24 h before experiment. Treatments were done to triplicate wells. Cells were treated with respective liposomes for 48 h. Following the termination of experiment cells were washed and promptly assayed for viability using MTT. Results were expressed as percent viability=[A550 (treated cells)-background/A550 (untreated cells)-background]×100.

## Appendix A Supplementary material

Supplementary material: **Fig. S1:** Transfection efficiency in cancer cells with different lipid and charge ratio. Cells were transfected using 0.3 µg pCMV-β-gal plasmid in SP lipoplex in different charge ratios (±, cationic lipid, *x*-axis). **Fig. S2:** DNA binding assay of lipoplex-associated plasmid DNA for SP liposomes after (A) 30 min (B) 4 h and (C) 24 h. The lipid: DNA charge ratios are indicated at the bottom of each well. SPD8, SPD4, SPD2, SPD1 represent lipid to DNA charge ratio of 8:1, 4:1, 2:1 and 1:1 respectively. DNA represents naked plasmid DNA. **Fig. S3:** Reverse transcriptase PCR studies of siRNA down-regulation in A549 cells. A, B and C represent 24 h, 48 h and 72 h treatment. Lane wise: Lane: (1) Untreated (2) 25 pmol MR (3) 50 pmol MR (4) 100 pmol MR (5) 25 pmol scrambled (6) 50 pmol scrambled (7) 100 pmol scrambled (8) 18 s untreated (9) 18s 25 pmol MR treated (10) 18 s 50 pmol MR treated (11) 18 s 100 pmol MR treated (12) 18 s 25 pmol Scrambled treated (13) 18 s 50 pmol Scrambled treated (14) 18 s 100 pmol Scrambled treated **Fig. S4:** Cytotoxicity studies in cells. MCF-7, A549, MDA-MB-231, CHO, HEK-293 and NIH-3T3 cells were transfected using 0.3 µg pCMV-β-gal plasmid (white bar) or with equal concentration of free spironolactone (spirono) (black bar) respectively for 48 h.

## Footnotes

Appendix A Supplementary data associated with this article can be found in the online version at doi:10.1016/j.dib.2016.01.003.
