# Supplementary material for: Data for stable formulation of steroid hormone receptor-targeted liposomes for cancer therapeutics
Source: Data Brief. 2016 Feb 27;7:428–31. doi: 10.1016/j.dib.2016.01.003 (PMC4786751; doi:10.1016/j.dib.2016.01.003)
Supplement: Supplementary file 1 — Supplementary material: Fig. S1: Transfection efficiency in cancer cells with different lipid and charge ratio. Cells were transfected using 0.3 µg pCMV-β-gal plasmid in SP lipoplex in different charge ratios (±, cationic lipid, x-axis). Fig. S2: DNA binding assay of lipoplex-associated plasmid DNA for SP liposomes after (A) 30 min (B) 4 h and (C) 24 h. The lipid: DNA charge ratios are indicated at the bottom of each well. SPD8, SPD4, SPD2, SPD1 represent lipid to DNA charge ratio of 8:1, 4:1, 2:1 and 1:1 respectively. DNA represents naked plasmid DNA. Fig. S3: Reverse transcriptase PCR studies of siRNA down-regulation in A549 cells. A, B and C represent 24 h, 48 h and 72 h treatment. Lane wise: Lane: (1) Untreated (2) 25 pmol MR (3) 50 pmol MR (4) 100 pmol MR (5) 25 pmol scrambled (6) 50 pmol scrambled (7) 100 pmol scrambled (8) 18 s untreated (9) 18s 25 pmol MR treated (10) 18 s 50 pmol MR treated (11) 18 s 100 pmol MR treated (12) 18 s 25 pmol Scrambled treated (13) 18 s 50 pmol Scrambled treated (14) 18 s 100 pmol Scrambled treated Fig. S4: Cytotoxicity studies in cells. MCF-7, A549, MDA-MB-231, CHO, HEK-293 and NIH-3T3 cells were transfected using 0.3 µg pCMV-β-gal plasmid (white bar) or with equal concentration of free spironolactone (spirono) (black bar) respectively for 48 h. [file mmc1.pdf]

AUTHOR DECLARATION

We wish to confirm that there are no known conflicts of interest associated with this publication and there has been no significant financial support for this work that could have influenced its outcome. We confirm that the manuscript has been read and approved by all named authors and that there are no other persons who satisfied the criteria for authorship but are not listed. We further confirm that the order of authors listed in the manuscript has been approved by all of us. We confirm that we have given due consideration to the protection of intellectual property associated with this work and that there are no impediments to publication, including the timing of publication, with respect to intellectual property. In so doing we confirm that we have followed the regulations of our institutions concerning intellectual property.

We understand that the Corresponding Author is the sole contact for the Editorial process (including Editorial Manager and direct communications with the office). He/she is responsible for communicating with the other authors about progress, submissions of revisions and final approval of proofs. We confirm that we have provided a current, correct email address which is accessible by the Corresponding Author and which has been configured to accept email from (pranav@hyderabad.bits-pilani.ac.in)

With Sincere regards

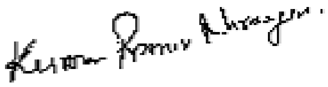

Place: Hyderabad

(Corresponding author)

Date: 23rd December, 2015

Kumar Pranav Narayan
Assistant Professor
Department of Biological Sciences
Birla Institute of Technology & Science, Pilani,
Hyderabad Campus
Hyderabad, India
