# Supplementary material for: Data for stable formulation of steroid hormone receptor-targeted liposomes for cancer therapeutics
Source: Data Brief. 2016 Feb 27;7:428–31. doi: 10.1016/j.dib.2016.01.003 (PMC4786751; doi:10.1016/j.dib.2016.01.003)
Supplement: Supplementary file 2 — Supplementary material [file mmc2.docx]

**Fig. S1:** Transfection efficiency in cancer cells with different lipid and charge ratio. Cells were transfected using 0.3µg pCMV-β-gal plasmid in SP lipoplex in different charge ratios (+/-, cationic lipid, x-axis).

**Fig. S2:** DNA binding assay of lipoplex-associated plasmid DNA for SP liposomes after (A) 30 min (B) 4 h and (C) 24 h. The lipid: DNA charge ratios are indicated at the bottom of each well. SPD8, SPD4, SPD2, SPD1 represent lipid to DNA charge ratio of 8:1, 4:1, 2:1 and 1:1 respectively. DNA represents naked plasmid DNA.

**Fig. S3:** Reverse transcriptase PCR studies of siRNA down-regulation in A549 cells. A, B & C represent 24 hours, 48 hours and 72 hours treatment. Lane wise: Lane: (1) Untreated (2) 25 pmol MR (3) 50 pmol MR (4) 100pmol MR (5) 25 pmol scrambled (6) 50 pmol scrambled (7) 100 pmol scrambled (8) 18s untreated (9) 18s 25pmol MR treated (10) 18s 50 pmol MR treated (11) 18s 100 pmol MR treated (12) 18s 25 pmol Scrambled treated (13) 18s 50 pmol Scrambled treated (14) 18s 100 pmol Scrambled treated

**Fig. S4:** Cytotoxicity studies in cells. MCF-7, A549, MDA-MB-231, CHO, HEK-293 and NIH-3T3 cells were transfected using 0.3µg pCMV-β-gal plasmid (white bar) or with equal concentration of free spironolactone (spirono) (black bar) respectively for 48 hours.

**Table S1:** Zeta size and zeta potential measurement for liposomes

**Table S2:** Zeta size and zeta potential measurement for lipoplexes

**
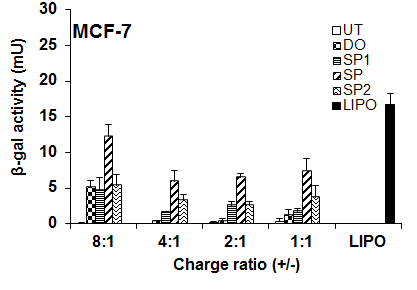
Fig. S1**

**Fig. S2**

**
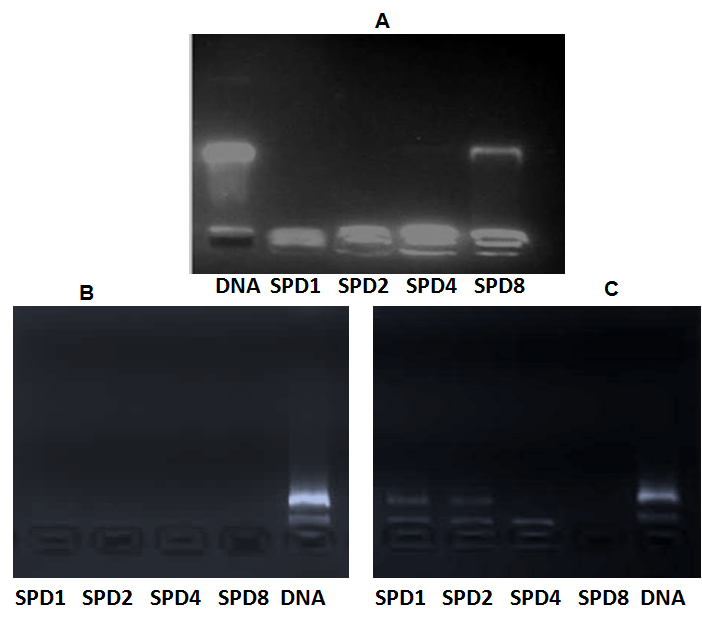
**

**Fig. S3**


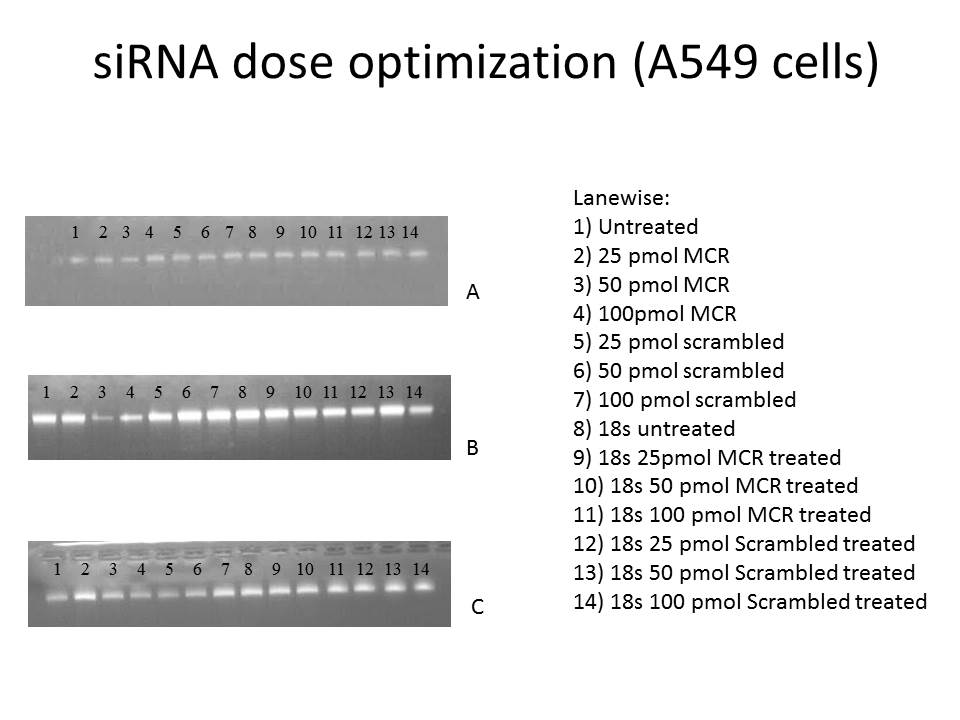


**Fig. S4**

**
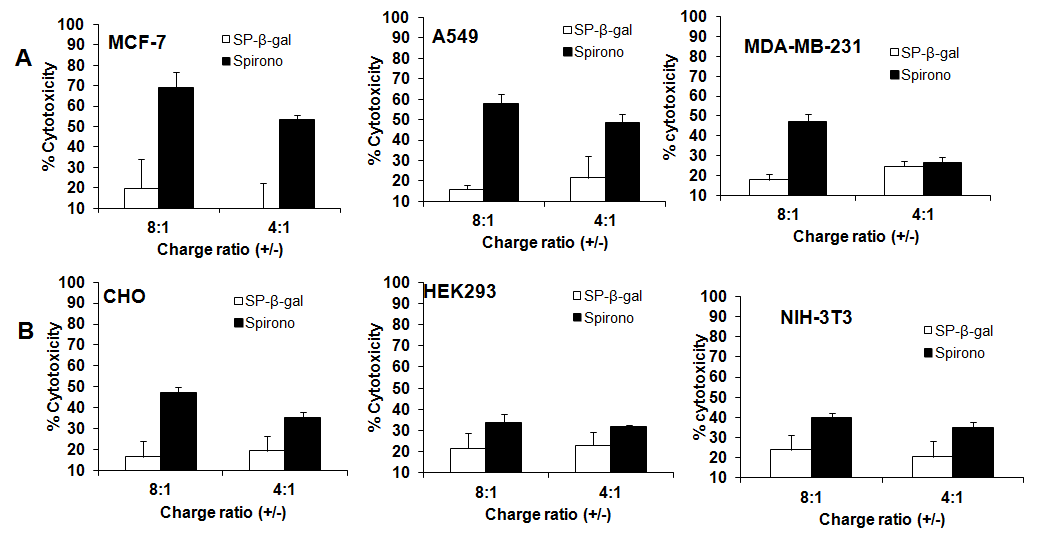
**

**Table S1**

| **Formulation** | **Time (in days)** | **Z Ave (nm)** | **Potential** |
| --- | --- | --- | --- |
| **DO** | **0** | **201.6** | **38.6** |
|  | **4** | **177.4** | **35.2** |
|  | **8** | **169.7** | **32.6** |
|  | **12** | **185.8** | **35.3** |
| **SP** | **0** | **288.1** | **23.7** |
|  | **4** | **289.9** | **25.7** |
|  | **8** | **290.4** | **21.5** |
|  | **12** | **293.3** | **17.4** |

**Table S2**

| **Formulation** | **Z Ave (nm)** | **Potential** |
| --- | --- | --- |
| **DO-DNA** | 503.8(±5.3) | 4.8(±1.0) |
| **SP-DNA** | 436.6(±15.9) | 4.8(±1.0) |
